# Mode of delivery and short-term infant health outcomes: a prospective cohort study in a peri-urban Indian population

**DOI:** 10.1186/s12887-018-1324-3

**Published:** 2018-11-06

**Authors:** Tamala Gondwe, Kalpana Betha, G. N. Kusneniwar, Clareann H. Bunker, Gong Tang, Hyagriv Simhan, P. S. Reddy, Catherine L. Haggerty

**Affiliations:** 1 Department of Epidemiology, Graduate School of Public Health, University of Pittsburgh, Pittsburgh, PA USA; 2 Department of Obstetrics and Gynecology, SHARE INDIA, MediCiti Institute of Medical Sciences, Medchal Mandal, Telangana State 501401 India; 3 Department of Community Medicine, SHARE INDIA, MediCiti Institute of Medical Sciences, Medchal Mandal, Telangana State 501401 India; 4 Department of Biostatistics, Graduate School of Public Health, University of Pittsburgh, Pittsburgh, PA USA; 5 Departments of Obstetrics, Gynecology, and Reproductive Sciences, School of Medicine, University of Pittsburgh, Pittsburgh, PA USA; 6 Magee-Womens Research Institute, Pittsburgh, PA USA

**Keywords:** Global health, India, Pediatrics, Respiratory infection, Diarrhea, Cesarean section, Epidemiology

## Abstract

**Background:**

Previous studies have found a relationship between cesarean section delivery and adverse outcomes in the offspring, partially attributing these findings to differential development of immunity in infants delivered by cesarean compared to vaginal delivery. The purpose of this study is to determine whether cesarean section delivery is associated with higher reports of adverse short-term infant health outcomes in a peri-urban Indian population.

**Methods:**

Data from a prospective pregnancy cohort study in a peri-urban region of Telangana State, India, were analyzed to assess the association between mode of delivery, cesarean section or vaginal, and maternal report of recent infant diarrhea and/or respiratory symptoms at a 6 month follow-up visit. Inverse probability weights were applied to log-binomial regression models to account for maternal pre-pregnancy, prenatal, and labor and delivery factors.

**Results:**

Of the 851 singleton infants delivered between 2010 and 2015, 46.7% were delivered by cesarean. Cesarean delivery was not associated with an increased report of infants having one or more of the outcomes (diarrhea, respiratory infection, or difficulty breathing) at 6 months (adjusted risk ratio 0.89, 95% confidence interval 0.76–1.03), nor was it associated with infants having a more severe outcome of comorbid diarrhea and respiratory infection (adjusted risk ratio 1.08, 95% confidence interval 0.58–2.04).

**Conclusion:**

Unlike findings in Western populations, in this peri-urban Indian population, cesarean delivery was not associated with higher reports of short-term adverse gastrointestinal or respiratory infant outcomes after accounting for pre-delivery maternal factors. Future research in this cohort could elucidate whether mode of delivery is associated with other adverse outcomes later in childhood.

**Electronic supplementary material:**

The online version of this article (10.1186/s12887-018-1324-3) contains supplementary material, which is available to authorized users.

## Background

Diarrhea and acute respiratory infections are among the leading preventable causes of mortality worldwide in children under age five years old, particularly in low and middle income countries [1]. Risk factors for these conditions include malnutrition, lack of breastfeeding, contaminated drinking water, and poor sanitation and hygiene practices [1]. While treatments such as antibiotics and oral rehydration therapy exist, primary prevention approaches such as those promoting a healthy immune system are arguably preferable.

Neonates are born with an almost sterile gastrointestinal tract which is then colonized by bacteria from their mother and birth environment [2]. As the intestinal microbiome plays an important role in generating host immune defense against pathogens, mode of delivery may influence both short and long term health among infants [2]. According to the ‘hygiene hypothesis’, cesarean section delivery increases the risk of immune related conditions in children when they are not initially exposed to their mother’s vaginal and intestinal microbes at birth, but instead are first exposed to microbes of the mother’s skin and the birth environment [3, 4]. This is thought to negatively impact the composition of the infant’s intestinal microbiome, impeding the development of a healthy immune system, and possibly leading to later adverse health outcomes [3, 4].

Observational, population-wide registry research studies have reported associations between cesarean delivery and offspring health conditions such as asthma, gastroenteritis, and atopic disease [5–7]. However, most of these studies have been conducted in high income countries. Less research has been conducted on the impact of mode of delivery on health outcomes in children in low and middle income countries, particularly within the first year of life.

The rate of cesarean delivery in India is high and increasing nationwide, with the most recent cesarean delivery estimate being 17.2% of all births in 2015, an increase from 8.5% in 2005 [8, 9]. Furthermore, an Indian study found that the fecal microflora of neonates at seven days old differed by mode of delivery [10]. Similar to prior studies, neonates delivered vaginally had bacteria that resembled the mother’s vaginal microbiome, whereas those delivered by cesarean had more bacteria associated with the hospital environment [10]. However, the impact of mode of delivery on short-term infant health outcomes in Indian populations is unknown. Determination of outcomes associated with cesarean delivery in this population is important, as it would call for programs to reduce the elevated cesarean rates and guide intervention programs among infants born by cesarean.

The purpose of this study is to identify whether infants in a peri-urban Indian population delivered by cesarean have higher reports of gastrointestinal (diarrhea), or respiratory (respiratory infection, or difficulty breathing) health problems at 6 months compared to infants delivered vaginally.

## Methods

### Study population

The Longitudinal Indian Family hEalth (LIFE) study is a prospective pregnancy cohort study of reproductive aged women residing in a peri-urban area in Telangana State, India. Details of the LIFE study design have been previously described [11]. Briefly, the LIFE study was established to assess the effect of the maternal environment on birth outcomes and child development. Between October 2009 and August 2011, 1227 women were enrolled pre-conception (80%) or in the first trimester of pregnancy (20%). Demographic information was collected at enrollment, details from labor and delivery abstracted from medical records, and follow-up data for mothers and children collected by anthropometric assessments and self-report on questionnaires. Follow-up questionnaires are administered at 1 month postpartum, and at 6 month intervals until the child is age two years, then annually thereafter.

Between March 2010 and December 2015, 1169 infants were born in the LIFE Study. Singleton infants with complete follow-up data through 6 months were included in this analysis, resulting in a sample of 851 of infants (73% of births through December 2015). Mothers of infants who did not have 6 month follow-up in December 2015 were not significantly different from mothers with follow-up in mode of delivery, caste/tribe, or working outside the home. However, more mothers who did not have 6 month follow-up were of the Hindu religion, reported a lower level of education, were younger at delivery (≤19 years old), and nulliparous compared to mothers with complete follow-up.

### Exposure and outcome variables

The primary exposure was mode of delivery (cesarean section or vaginal delivery) as recorded on the LIFE study labor and delivery abstraction form. Vaginal deliveries included spontaneous vaginal deliveries (*n* = 428), vaginal deliveries with forceps assist (*n* = 23), vaginal deliveries with vacuum assist (n = 2), or vaginal deliveries with breech extraction (*n* = 1).

Outcomes were assessed at the 6 month follow-up visit and include maternal report of infant respiratory infection (cough or cold) in the past month, infant diarrhea in the past month, or infant signs and symptoms of difficulty breathing in the past 3 months. We assessed two combinations of these outcomes. First, we considered a composite representing one or more of the health outcomes. Second, we categorized an outcome to include both diarrhea and respiratory infection, which we considered to represent more severe morbidity.

### Statistical analysis

Bivariate analyses were conducted to calculate the crude relationships between mode of delivery and infant outcomes using log-binomial regression models. In addition, infant anthropometric assessments, maternal report of infant birth factors, infant feeding, and household hygiene and sanitation practices were also assessed for their association with the outcomes.

Propensity score adjustment using inverse probability weights (IPWs) was applied to minimize bias and to balance the exposure groups by pre-delivery factors. To estimate the propensity score, a logistic regression model with mode of delivery as the outcome and pre-delivery factors as predictors was used to obtain a probability of delivery by cesarean. These factors encompassed factors pre-pregnancy, prenatal, and during labor and delivery. Common support of the propensity score model was visually evaluated by comparing box plots of the propensity score distribution, and balance of the propensity score model was assessed by calculating standardized bias (Additional file 1: Table S1) [12].

The inverse of the estimated propensity score was incorporated as a weight in the log-binomial regression models to assess the adjusted association between mode of delivery and infant outcomes, obtaining adjusted relative risk (aRRs) and 95% confidence intervals (CIs). Secondary analyses were conducted adjusting for postpartum covariates including infant sex, timing of breastfeeding initiation (< 1 h vs > 2 h), and infant waste disposal method. All analyses were conducted using SAS 9.3 (SAS Institute, Cary, NC).

## Results

### Population characteristics

Of the 851 infants with 6 month follow-up in the LIFE study, 46.7% were delivered by cesarean section. Of these, 418 (49.1%) were reported to have had one or more of the outcomes (diarrhea, respiratory infection, or difficulty breathing), and 45 (5.3%) had both diarrhea and respiratory infection in the past month. Differences in characteristics and exposures for infant outcome groups are presented in Table 1.

**Table 1:** Characteristics associated with report of infant outcomes at six month follow-up in the LIFE study

		Diarrhea or Respiratory Infection and/or Difficulty Breathing		*p*-value	Comorbid Diarrhea and Respiratory Infection		*p*-value
		Yes	No		Yes	No	
		%	%		%	%	
		*N* = 418	*N* = 433		*N* = 45	*N* = 806	
Infant Characteristics							
Infant sex	Boy	53.5	51.1	0.47	38.6	53.0	0.06
	Girl	46.5	48.9		61.4	47.0	
Gestational age at birth	Mean weeks ± SD	38.9 ± 2.4	38.8 ± 2.5	0.93	38.9 ± 2.4	38.6 ± 2.5	0.48
Birth weight	Mean kg ± SD	2.8 ± 0.4	2.8 ± 0.5	0.46	2.8 ± 0.4	2.7 ± 0.5	0.21
Weight at 6 months	Mean kg ± SD	7.1 ± 1.0	7.1 ± 1.1	0.95	7.1 ± 1.0	6.9 ± 1.0	0.09
Age at time of questionnaire	Mean months ± SD	6.5 ± 1.0	6.5 ± 0.8	0.90	6.5 ± 0.9	6.6 ± 0.8	0.63
Infant Feeding							
Ever breastfed	Yes	100	99.5	0.17	100	99.8	0.74
Time to breast feeding initiation from birth (hours)	≤1 h	70.3	69.6	0.81	57.8	70.6	0.07
	2–20 h	29.7	30.4		42.2	29.4	
Breastfeeding at 6 months	Yes	98.1	97.7	0.68	100	97.7	0.31
Given anything other than breast milk to infant	Yes	87.8	90.3	0.25	82.2	89.4	0.13
Hygiene/ Sanitation							
Where is your infants waste disposed?	Put/rinsed into toilet or latrine	23.5	20.3	0.02	37.8	21.0	0.004
	Put/ rinsed into open drain or ditch	31.6	25.6		37.8	28.0	
	Buried	19.9	29.0		4.4	25.7	
	Thrown into garbage	10.4	12.4		11.1	11.5	
	Left in the open	14.6	12.7		8.9	13.9	
Maternal Health at 6 months							
Ailments in the past month	Maternal Diarrhea	0.7	0	0.08	2.3	0.2	0.03
	Maternal Respiratory Infection	2.4	0.5	0.02	11.4	0.9	< 0.0001

For both outcome groups, there were no significant differences in infant sex, gestational age, birth weight, weight at 6 months, or infant age at the time of questionnaire, although a higher proportion of girls had comorbid diarrhea and respiratory infection as compared to boys (*p* = 0.06). Breastfeeding was nearly universal in this population, and over 98% of mothers were still breastfeeding at the 6 month follow-up. Rates of breastfeeding did not differ by infant outcome, although a greater proportion of infants with comorbid diarrhea and respiratory infection had report of later initiation of breastfeeding (≥2 h after delivery vs ≤1 h after delivery) (*p* = 0.07). Moreover, more infants with one or more outcomes reported a higher number of supplemental feedings per day.

Among analyses of hygiene and sanitation factors, a higher proportion of mothers of infants who had one or more outcomes reported disposing of their infants’ waste by rinsing the diaper in the toilet/latrine (23.5% vs 20.3% of mothers whose infants did not have any illness) or an open ditch (31.6% vs 25.6%), or leaving it in the open (14.6% vs 12.7%), while a smaller proportion buried the waste (19.9% vs 29%) or disposed into the garbage (10.4% vs 12.4%). A higher proportion of mothers of infants who had both diarrhea and respiratory infection reported disposing of their infants’ waste by rinsing the diaper in the toilet/latrine (37.8% vs 21.0%) or an open ditch (37.8% vs 28.0%), while a smaller proportion buried (4.4% vs 25.7%) or left it in the open (8.9% vs 13.9%).

Three mothers reported having diarrhea in the past month, and 12 reported having respiratory infection in the past month. More infants with one or more outcomes had a mother reporting diarrhea (2.4% vs 0.5%), and a higher proportion of infants with comorbid diarrhea and respiratory infection had mothers with both of these ailments (2.3% vs 0.2% maternal diarrhea, and 11.4% vs 0.9% and maternal respiratory infection). However, temporality of infection (mother infected first or infant infected first) could not be established.

### Regression analysis

In the unadjusted log-binomial regression model predicting one or more of the outcomes, cesarean delivery was associated with a 12% reduced risk of the infant subsequently experiencing one or more of the outcomes, although the relationship was of borderline statistical significance (RR 0.88, 95% CI 0.77–1.01, Table 2). The relative risk was not attenuated in the IPW log-binomial model, (aRR 0.89, 95% CI 0.76–1.03). In secondary analysis adjusting for the IPW and infant sex, hours after birth that breastfeeding was initiated, and infant waste disposal method as covariates, the association did not change (aRR 0.88, 95% CI 0.76–1.03) (Additional file 2: Table S2).

**Table 2:** The association between mode of delivery and infant health outcomes at 6 month follow-up in the LIFE study, adjusting for pre-delivery maternal factors

	≥1 outcome at 6 months (Diarrhea, difficulty breathing, or respiratory infection)		Comorbid diarrhea and respiratory infection at 6 months	
	Unadjusted RR (95% CI)	Weighted^a^ aRR (95% CI)	Unadjusted RR (95% CI)	Weighted^a^ aRR (95% CI)
Cesarean vs Vaginal delivery	0.88 (0.77–1.01)	0.89 (0.76–1.03)	0.91 (0.52–1.62)	1.08 (0.58–2.04)

^a^Variables incorporated in weight: pre-pregnancy BMI, parity, education; first trimester prenatal vitamin use, diagnosis of feet swelling during third trimester, unable to perform regular duties due to illness/injury during third trimester, prenatal vaginal bleeding; age at delivery, and one or more labor and delivery complications

Similarly, the log-binomial models of mode of delivery predicting concomitant diarrhea and respiratory infection were not statistically significant in unadjusted (RR 0.91, 95% CI 0.52–1.62), or IPW adjusted analysis (aRR 1.08, 95% CI 0.58–2.04, Table 2). Additional adjustment for infant sex, hours after birth that breastfeeding was initiated, and infant waste disposal method in secondary analysis did not significantly change the results (aRR 0.94, 95% CI 0.48–1.85) (Additional file 2: Table S2).

## Discussion

In this peri-urban Indian population, infants delivered by cesarean were not found to have a higher risk of reported adverse gastrointestinal or respiratory health outcomes assessed at 6 months of age as compared to those delivered vaginally. Although IPW adjusted analyses were not statistically significant, our findings suggested a marginally lower risk of respiratory infection, difficulty breathing, or diarrhea, but a modestly increased risk of comorbid respiratory infection and diarrhea in infants delivered by cesarean compared to vaginal delivery. Accounting for infant sex, hygiene, and breastfeeding covariates nullified these associations.

Our study focused on assessing short-term infant health and did not find a significant association between mode of delivery and respiratory or gastrointestinal health problems after accounting for pre-delivery maternal factors in our analysis. Studies in similar demographic populations in Malaysia, Iraq, and Brazil have focused on the association of cesarean delivery on asthma in children ranging in age from six to 15 years, and have also not found a statistically significant association. Rather, other factors such as family history of allergic disease, parental education, crowding in the home, and exposure to cigarette smoke were found to be predictive of the adverse health outcomes [13–15]. Similar to our results, these findings suggest that children in low and middle income countries have a range of exposures that can lead to adverse respiratory health outcomes other than mode of delivery.

Conversely, a longitudinal study conducted in both India and Vietnam found that the likelihood for caregiver reported asthma at age eight years was twice as high for those delivered by cesarean compared to those vaginally delivered, after accounting for socio-demographic risk factors in multivariable analysis [16]. This finding was similar to studies in Western countries, which have found an increased association between mode of delivery and adverse infant health outcomes. Using a Swedish population-based registry to assess the association between mode of delivery with asthma and gastroenteritis, one study found that hospital admission for asthma or gastroenteritis in children at least a year old was increased in those delivered by cesarean [5]. The link between cesarean delivery and gastrointestinal symptoms in the first year of life was also found in a German cohort, and in infants born preterm in an Australian population based study [17, 18].

The rate of breastfeeding has been identified as an important predictor of infant outcomes in previous studies, and initiation of breastfeeding is generally lower in pre-labor cesarean deliveries [19]. In populations with universal breastfeeding through 6 months, such as ours, the effect of mode of delivery on infant health may be negligible, as breast milk also has immune building properties [18]. A study of the microbes in breast milk samples of Canadian women found that breast milk contained the beneficial *Lactobacillus* bacteria regardless of mode of delivery [20]. Thus, in our population with high breast feeding rates, 99% ever breastfed and over 97% still breastfeeding at the six month follow-up, even infants delivered by cesarean could receive beneficial microbes to promote development of a healthy immune system via breast feeding.

Regarding mode of delivery, while the rate of 46.7% cesarean delivery in our study population appears high compared to the national rate of 17.2% in 2015, this rate mirrors the estimated cesarean delivery rate of 58% in Telangana State in 2015 [21]. This shows that there is wide variation in the cesarean delivery rate in India by geographic location. Some of the proposed reasons for the increasing cesarean delivery rate in India include a growing maternal preference for medicalized deliveries, in addition to convenience and financial profit on the part of the medical provider [22, 23]. Further research is needed to identify whether the increase in cesarean delivery rates in India is due to medical necessity, and whether it justifies exceeding the World Health Organization’s proposed rate of 5 to 15% of births [24].

One of the major strengths of our study is that we were able to control for the effect of pre-delivery maternal factors that may have biased a woman towards cesarean delivery. In addition, as this is a prospective study we are able to assess short-term health outcomes in a large sample of over 800 infants, which offers a unique perspective in the literature that is dominated by the assessment of mode of delivery in older children in Western countries.

By creating composite variables for the adverse respiratory and gastrointestinal outcomes we could assess combinations of both common and more severe outcomes. However, unlike our exposure which is verified on medical records, the outcomes were determined by maternal report and were not clinically confirmed. Thus, outcome misclassification is possible. While our study was limited to the assessment of short- term infant outcomes, future studies in our population can consider both short-term and longer term childhood outcomes, including asthma. There is likely unmeasured confounding of predictors that we could not assess, including parental and family history of asthma or atopic disease. In addition, differences in maternal characteristics by religion, education, age, and parity in mothers with and without follow-up through 6 months may have affected our results. These differences may reflect the cultural practices of a woman relocating from her marital home to her mother’s residence when delivering a first child [11]. Lastly, onset of labor was not measured in our study; therefore, some infants delivered by cesarean may have been exposed to their mother’s vaginal microbiota during a period of labor, which would negate the ‘hygiene hypothesis’. Additional studies in low and middle income settings are needed to assess the causal associations between mode of delivery, and infant respiratory or gastrointestinal health outcomes, accounting for timing of labor.

## Conclusion

Our study shows that mode of delivery may not significantly impact infant health outcomes in peri-urban settings in India, and possibly other low and middle income countries. While research in urban, Western countries has found an association between mode of delivery and adverse infant outcomes, our study suggests that other factors including infant sex, mother’s health, and sanitation/hygiene perhaps play a more significant role in infant respiratory and gastrointestinal health in similar settings.

## Additional files

Additional file 1: **Table S1.** Standardized Bias.pdf provides a comparison of standardized bias for pre-delivery maternal variables before and after weighting by the propensity score. (PDF 197 kb)

Additional file 2: **Table S2.** Infant health outcomes secondary analysis.pdf shows findings after additional adjustment for infant sex, breastfeeding initiation, and hygiene factors. (PDF 250 kb)

## Abbreviations

aRR: Adjusted relative risk
CI: Confidence interval
IPW: Inverse probability weight
LIFE: Longitudinal Indian Family hEalth study
RR: Relative risk
