# Supplementary material for: Mode of delivery and short-term infant health outcomes: a prospective cohort study in a peri-urban Indian population
Source: BMC Pediatr. 2018 Nov 6;18:346. doi: 10.1186/s12887-018-1324-3 (PMC6220445; doi:10.1186/s12887-018-1324-3)
Supplement: Supplementary file 1 — Table S1. Standardized Bias.pdf provides a comparison of standardized bias for pre-delivery maternal variables before and after weighting by the propensity score. (PDF 197 kb) [file 12887_2018_1324_MOESM1_ESM.pdf]

Table S1 Maternal pre-delivery characteristics by mode of delivery and standardized bias before and after weighting

Characteristic	Vaginal Delivery n=454	Cesarean Delivery n=397	Unweighted Standardized Bias†	Weighted Standardized Bias†
	n(%)	n(%)		
Pre-pregnancy				
BMI (Mean kg/m^2 (Std. Dev))	19.5 (2.9)	20.6 (3.6)	0.34	-0.04
Multiparous	349 (81)	286 (78)	-0.09	-0.03
Level of education				
None	68 (15)	46 (12)	0.23	0.09
Primary	126 (28)	88 (22)		
Secondary	227 (50)	213 (54)		
Higher education	33 (7)	50 (13)		
Prenatal				
First trimester prenatal vitamin use	111(26)	152(42)	0.33	-0.05
Diagnosed with feet swelling during third trimester	25(5.7)	37(9.7)	0.15	-0.12
Not able to do regular duties due to illness/injury during third trimester	8(1.8)	17(4.5)	0.15	0.05
Prenatal vaginal bleeding	5(1.2)	14(3.8)	0.17	-0.17
Delivery				
Age at delivery	22.9 (2.9)	23.5 (3.1)	0.20	-0.05
One or more labor and delivery complications	42(9.3)	142(36)	0.67	-0.05

† Standardized bias = difference in means or proportions divided by standard error; imbalance defined as an absolute value >0.25
