# Supplementary material for: Mode of delivery and short-term infant health outcomes: a prospective cohort study in a peri-urban Indian population
Source: BMC Pediatr. 2018 Nov 6;18:346. doi: 10.1186/s12887-018-1324-3 (PMC6220445; doi:10.1186/s12887-018-1324-3)
Supplement: Supplementary file 2 — Table S2. Infant health outcomes secondary analysis.pdf shows findings after additional adjustment for infant sex, breastfeeding initiation, and hygiene factors. (PDF 250 kb) [file 12887_2018_1324_MOESM2_ESM.pdf]

Table S2 The association between mode of delivery and infant health outcomes at six month follow-up in the LIFE study, adjusting for pre-delivery maternal factors and postpartum covariates

	≥1 outcome at 6 months (Diarrhea, difficulty breathing, or respiratory infection)		Comorbid diarrhea and respiratory infection at 6 months	
	Unadjusted RR (95% CI)	Weighted * aRR (95% CI)	Unadjusted RR (95% CI)	Weighted * aRR (95% CI)
Cesarean vs Vaginal delivery	0.88 (0.77-1.01)	0.88 (0.76-1.03)	0.91 (0.52-1.62)	0.94 (0.48-1.85)
Infant sex: girl vs boy	0.95 (0.83-1.09)	0.91 (0.78-1.06)	1.74 (0.96-3.14)	1.71 (0.84-3.48)
Infant breastfed 2+ hours after birth vs <1 hour	0.99 (0.85-1.15)	0.87 (0.74-1.03)	1.87 (1.03-3.37)	1.55 (0.79-3.03)
Diaper/Waste disposal method				
Put/rinsed into toilet or latrine	1.17 (0.90- 1.52)	1.17 (0.88-1.56)	1.76 (0.67-4.64)	1.57 (0.48-5.18)
Put/ rinsed into open drain or ditch	1.20 (0.94- 1.55)	1.19 (0.90- 1.56)	1.35 (0.51-3.57)	1.39 (0.43-4.49)
Buried	0.88 (0.67- 1.16)	0.78 (0.57- 1.06)	0.18 (0.04-0.93)	0.09 (0.008-1.15)
Thrown into garbage	Ref.	Ref.	Ref.	Ref.
Left in the open	1.16 (0.88- 1.55)	1.12 (0.81- 1.54)	0.67 (0.18-2.42)	0.67 (0.14-3.09)

* Variables incorporated in weight: pre-pregnancy BMI, Parity, Level of education; First trimester prenatal vitamin use, Diagnosed with feet swelling during third trimester, Not able to do regular duties due to illness/injury during third trimester, Prenatal vaginal bleeding; Age at delivery, one or more labor and delivery complications
